# Succinate Accumulation Links Mitochondrial MnSOD Depletion to Aberrant Nuclear DNA Methylation and Altered Cell Fate

**Published:** 2020-12-09

**Authors:** Kimberly L. Cramer-Morales, Collin D. Heer, Kranti A. Mapuskar, Frederick E. Domann

**Affiliations:** 1 Department of Radiation Oncology, The University of Iowa, Iowa City, Iowa 52242, USA; 2 Department of Surgery, The University of Iowa, Iowa City, Iowa 52242, USA; 3 Department of Pathology, The University of Iowa, Iowa City, Iowa 52242, USA; 4 Holden Comprehensive Cancer Center, The University of Iowa, Iowa City, Iowa 52242, USA

**Keywords:** Mitochondria, Superoxide dismutase, Iron homeostasis, Succinate dehydrogenase, Retrograde signaling, DNA methylation, Epigenetic control, Gene expression

## Abstract

Previous studies showed that human cell line HEK293 lacking mitochondrial superoxide dismutase (MnSOD) exhibited decreased succinate dehydrogenase (SDH) activity, and mice lacking MnSOD displayed significant reductions in SDH and aconitase activities. Since MnSOD has significant effects on SDH activity, and succinate is a key regulator of TET enzymes needed for proper differentiation, we hypothesized that *SOD2* loss would lead to succinate accumulation, inhibition of TET activity, and impaired erythroid precursor differentiation. To test this hypothesis, we genetically disrupted the *SOD2* gene using the CRISPR/Cas9 genetic strategy in a human erythroleukemia cell line (HEL 92.1.7) capable of induced differentiation toward an erythroid phenotype. Cells obtained in this manner displayed significant inhibition of SDH activity and ~10-fold increases in cellular succinate levels compared to their parent cell controls. Furthermore, *SOD2*^−/−^ cells exhibited significantly reduced TET enzyme activity concomitant with decreases in genomic 5-hmC and corresponding increases in 5-mC. Finally, when stimulated with δ-aminolevulonic acid (δ-ALA), *SOD2*^−/−^ HEL cells failed to properly differentiate toward an erythroid phenotype, likely due to failure to complete the necessary global DNA demethylation program required for erythroid maturation. Together, our findings support the model of an SDH/succinate/TET axis and a role for succinate as a retrograde signaling molecule of mitochondrial origin that significantly perturbs nuclear epigenetic reprogramming and introduce MnSOD as a governor of the SDH/succinate/TET axis.

## Introduction

Epigenetic processes provide a mechanism for integrating metabolic inputs and translating them into various phenotypic outputs [1,2]. Indeed, epigenetic modifiers often require or are inhibited by key citric acid cycle (CAC) metabolites (*e.g*. succinate, fumarate, alpha-ketoglutarate, acetyl-CoA) and co-factors (*e.g*. NAD^+^, FAD) [3]. One such metabolite, succinate, has recently been shown to act as a mitochondrial retrograde signal that exerts effects on the epigenome *via* inhibition of histone demethylases and the ten-eleven translocase (TET) family of DNA demethylase enzymes [4,5].

TET family enzymes contribute to the maintenance of the epigenome by catalyzing the oxidation of 5-methylcytosine (5-mC) to 5-hydroxymethylcytosine (5-hmC), initiating a series of oxidation reactions necessary for the removal of 5-mC. Like other epigenetic modifiers, TET family enzymes are sensitive to metabolic perturbations affecting the absolute and relative concentrations of their co-factors and products. Their required co-factors and substrates are identical to those of the collagen and HIF1 proline hydroxylase (PHD) family of enzymes and include molecular oxygen (O_2_), ferrous iron (Fe^2+^), α-ketoglutarate (α-KG), and ascorbate [6]. Using an Fe^IV^-oxo complex generated by the oxidative decarboxylation of α-KG, TET enzymes catalyze the abstraction of a hydrogen from 5-mC, which is then hydroxylated. In the process, the oxidative decarboxylation of α-KG yields succinate and carbon dioxide (CO_2_) [7]. Through a product-induced negative-feedback mechanism, the inhibition of TET enzymes by succinate allows these enzymes to respond directly to changes in cellular metabolism, particularly to nodes linked to succinate and α-KG production and consumption.

Succinate and α-KG serve as key regulators of self-renewal and differentiation through TET enzymes. However, this regulation is complex and context-dependent [8,9]. For example, naïve embryonic stem cells sustain a high α-KG: succinate ratio to facilitate DNA and histone demethylation required for the maintenance of pluripotency [10,11]. However, α-KG is also important for the facilitation of epigenetic remodeling during differentiation of primed pluripotent stem cells [12] and premalignant cells in which p53 has been activated [13]. In the case of erythroid precursors, the model used in this study, global demethylation is required for the differentiation into red blood cells (RBCs) [14] and loss of TET enzymatic activity results in promoter hypermethylation and impaired differentiation of embryonic and hematopoietic stem cells [12,15,16]. Thus, the metabolic ratio of succinate to α-KG is a key factor in determining self-renewal and differentiation through modulation of TET activity.

A primary regulator of succinate levels is the activity of succinate dehydrogenase (SDH). SDH is a 4-subunit complex located in the inner membrane of the mitochondria that plays a role in the CAC *via* catalysis of the oxidation of succinate to fumarate as well as serving the role of Complex II in the electron transport chain by passing electrons from succinate or FADH_2_ directly to coenzyme Q in the mitochondrial inner membrane. Pharmacological inhibition or genetic mutation of SDH leads to accumulation of succinate and inhibition of α-KG-dependent enzymes such as TETs [5,17–19]. SDH activity can also be modulated by reactive oxygen species (ROS). Specifically, superoxide (O_2_^•−^) can inactivate iron-sulfur (Fe-S) cluster-containing enzymes such as SDH [18,19], as well as other enzymes in the CAC (aconitase, fumarate hydratase) [20,22,23], heme synthesis (ferrochelatase; FECH) [24], and amino acid metabolism [25,26] among several others.

Manganese superoxide dismutase (MnSOD) catalyzes the dismutation of mitochondrial O_2_^•−^ to hydrogen peroxide (H_2_O_2_). Cells lacking MnSOD exhibit elevated steady state levels of O_2_^•−^ and reduced SDH activity, likely due to inactivation of Fe-S clusters [20,27–29]. Since succinate is a direct regulator TET enzymatic activity and MnSOD has significant effects on SDH activity, we hypothesized that MnSOD loss would lead to succinate accumulation, inhibition of TET enzyme activity and altered cellular phenotype. To test this hypothesis, we used the CRISPR/Cas9 strategy to genetically disrupt the *SOD2* gene in two different human cell lines, human embryonic kidney 293 (HEK293) generated in a previous study [28], and a human erythroleukemia (HEL) generated in this study, thereby eliminating MnSOD activity. We chose HEL cells as a model because RBC maturation to produce mature enucleated erythrocytes requires rapid and widespread demethylation of genomic DNA [14] and these cells can be induced to differentiate toward mature red cells upon supplementation with δ-aminolevulinic acid (δ-ALA). Thus, we could utilize this model to study the effects of MnSOD loss on DNA demethylation-dependent erythroid differentiation.

Following characterization of the genetically disrupted *SOD2* cell lines, we measured the activities of mitochondrial enzymes sensitive to inactivation by O_2_^•−^ (SDH, aconitase), succinate levels, TET enzyme activity, TET substrate (5-mC) and product (5-hmC) levels, and ability of HEL cells to undergo erythroid maturation in response to δ-ALA exposure. We found that MnSOD loss resulted in decreased SDH activity, succinate accumulation, reduced activity of TET enzymes, inhibition of DNA demethylation, and impaired erythroid differentiation. Taken together, our results introduce a mechanism through which MnSOD exerts epigenetic control over differentiation through an MnSOD/SDH/succinate/TET axis, and further support a role for succinate as a retrograde signaling molecule of mitochondrial origin.

## Materials and Methods

### Cell lines

HEK293T cells were obtained from American Type Culture Collection (ATCC) maintained in DMEM supplemented with 10% fetal bovine serum in a humidified 37°C incubator with 5% CO_2_ and were routinely sub-cultured before reaching confluence by detachment with TrypLE Express (Invitrogen, Carlsbad, CA).

HEL 92.1.7 cells were obtained from ATCC and cultured in RPMI1640 supplemented with 10% fetal bovine serum in a humidified 37°C incubator with 5% CO_2_ and were routinely sub-cultured by 1:10 dilutions in fresh media.

### Plasmid construction and transfection

The pD130-GFP expression vector was designed to contain expression cassettes for green fluorescent protein (GFP), Cas9 endonuclease, and a CRISPR chimeric cDNA with the gRNA moiety designed to target exon 3 of *SOD2* [ATATCAATCATAGCATTTTC]. This plasmid was custom synthesized (DNA2.0, Menlo Park, CA) and following amplification, purification, and characterization was used to transfect the two cell lines described above.

HEK293T cells were transiently transfected by calcium phosphate precipitation. Five days after transfection GFP+ cells were sorted and collected by flow cytometry and colonies were selected from single clones using cloning discs.

HEL 92.1.7 cells were transfected by electroporation at various conditions using the Gene Pulser Xcell^™^ Electroporation System (BioRad, USA). In brief, 5 × 10^6^ cells were suspended in 500 μl complete RPMI medium, mixed with 30 μg of pD130-GFP-plasmid in a 4 mm electroporation cuvette, and incubated on ice for 10 min. After electroporation with a single pulse (electrical parameters of 300 V, 950 μF) the cells were transferred into complete RPMI medium at a density of 1 × 10^6^ cells per ml and returned to the incubator. Five days after transfection GFP+ cells were sorted by flow cytometry and colonies were selected from single clones seeded in 96-well plates.

### Western blot analyses

Western blots were performed as previously described [26]. Briefly, total proteins were extracted from HEL 92.1.7 clones with standard RIPA buffer plus protease inhibitors and quantified by Bradford assay. Proteins were separated by SDS-PAGE, transferred to nitrocellulose membranes and probed with primary antibodies detecting MnSOD (Cat # 06–984, Millipore, Billerica, MA) and beta-tubulin (University of Iowa Hybridoma Core, Iowa City, IA).

### Aconitase activity

Aconitase activity was measured as described previously [30]. In brief, in a spectrophotometric assay where total cellular protein was combined with NADP^+^ and isocitrate dehydrogenase and the appearance of NADPH was monitored at 340 nm every 30s for 5 minutes.

### Succinate dehydrogenase histochemistry

Succinate dehydrogenase activity visualized via NBT reduction histochemistry. Culture medium was aspirated and cells were washed twice with PBS. Cell culture dishes were allowed to air dry at room temperature. A solution containing 0.55 mM NBT and 0.05 M sodium succinate was applied and the dishes were incubated overnight at 37°C and washed again with PBS before images were captured.

### Determination of succinate levels

Succinate levels were determined using a colorimetric assay (Sigma-Aldrich Cat # MAK184) according to the manufacturer’s specifications.

### Tet DNA demethylase activity and 5-hydroxymethylcytosine (5hmC) measurement

Cellular TET DNA demethylase activities were assessed using the EpiGentek TET activity assay platform. Total genomic 5-methylcytosine and 5-hydroxymethylcytosine levels were measured in genomic DNA with the corresponding immunoassay systems also from EpiGentek according to the manufacturer’s instructions.

### Flow cytometry analysis

Determinations were performed with a flow cytometer (FACScan, Becton Dickinson, San Jose, CA). HEK293T or HEL 92.1.7 cells were gated on the basis of their forward scatter and side scatter signals with logarithmic scales. The region representing single, non-aggregated cells was gated before each experience with non-agglutinated samples to count events in the single-cell region. Fluorescence profiles were collected only on those cells appearing in this region to exclude agglutinates and debris. For each sample, 20,000 events were collected in logarithmic scale. Photomultiplier tube voltage for the fluorescence detector was adjusted before running samples to obtain a negative fluorescence signal for control (untransfected) samples. This source power value was fixed for all measurements. Computer software (WinMDI, version 2.8, Windows multiple document interface, flow cytometry application, Microsoft, Redmond, WA) was used for data analysis to calculate mean fluorescence intensity (MFI) from fluorescence histograms.

Antibodies utilized were directed against TFRC (PE-tagged anti-CD71 clone M-A712, BD Biosciences, San Jose, CA) and Glycophorin A (PE-tagged-anti-CD235a clone REA175, Miltenyi Biotech, Auburn, CA), markers of human erythroid maturation. Cells were incubated with antibodies under conditions previously described [31–33] and flow cytometry was performed as described above.

## Results

### Characterization of MnSOD loss in CRISPR/Cas9-edited HEL 92.1.7 cells

**Figure 1:**
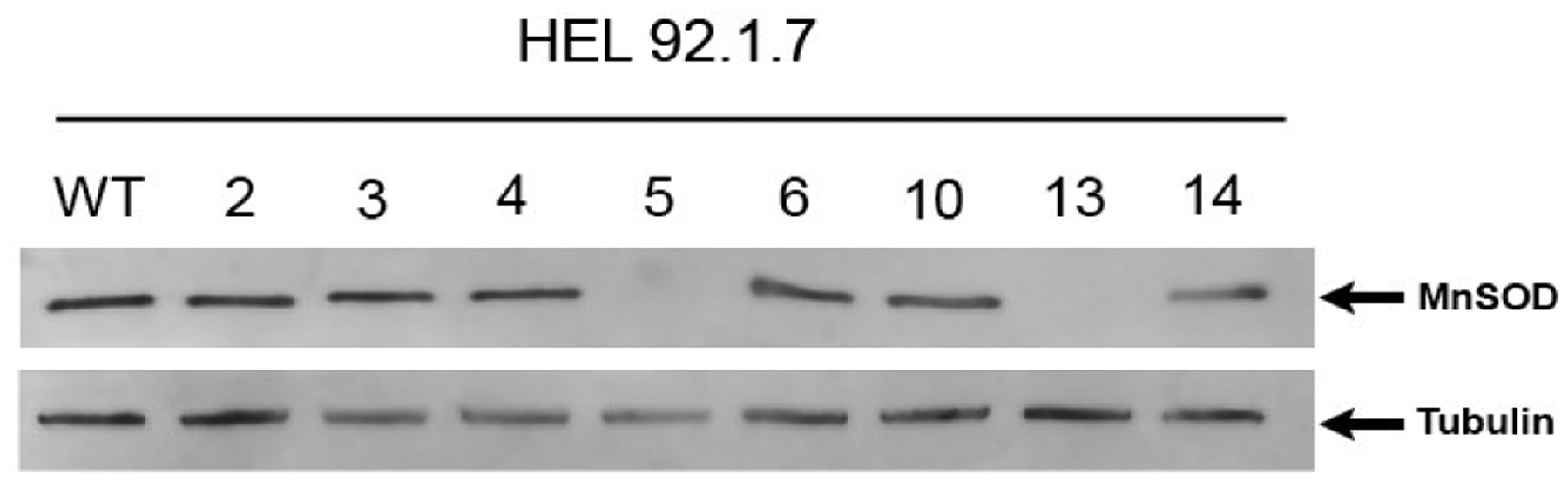
Characterization of MnSOD loss in CRISPR/Cas9-edited HEL 92.1.7 cells. The CRISPR/Cas9 system for creating specific genetic deletions was used to target exon 3 of human *SOD2* in a human erythroleukemia cell line (HEL 92.1.7). Total protein was isolated from clones transfected with the CRISPR/Cas9 expression construct and subjected to Western blot analysis. Two clones were confirmed to show absence of MnSOD protein expression by Western blot analysis.

The CRISPR/Cas9 system for creating specific genetic deletions was used to target exon 3 of human *SOD2* in a HEL 92.1.7 cells. *SOD2*^*−/−*^ HEK293T were generated previously [28]. Two clones from each cell line were confirmed to show absence of MnSOD protein expression by Western blot analysis (Figure 1) [28]. In a previous study, we showed that MnSOD-deficient 293T (*SOD2*^*−/−*^) cells showed significantly increased DHE and MitoSOX fluorescence compared to wild-type (WT) cells, suggesting increased cytosolic and mitochondrial pro-oxidants levels, respectively [28].

### SOD2 KO cells exhibited decreased activity in enzymes sensitive to inactivation by O_2_^•−^ and accumulation of cellular succinate

**Figure 2:**
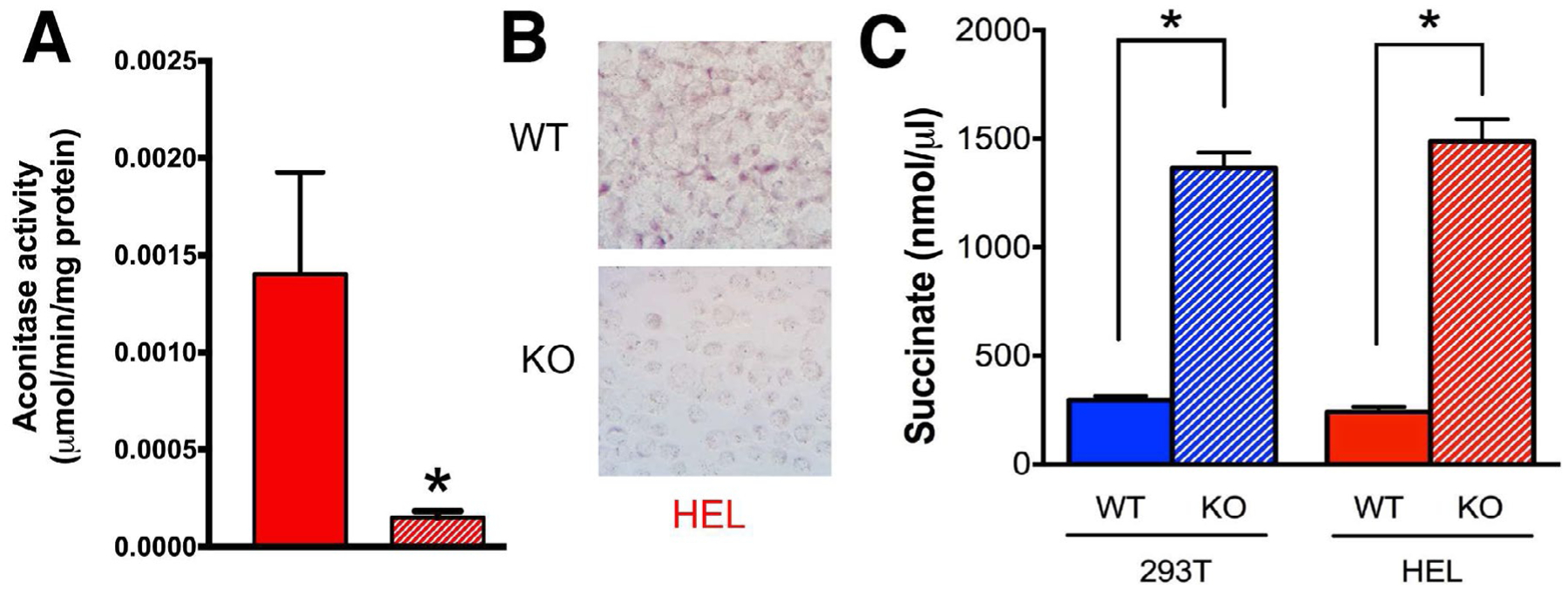
*SOD2* KO cells exhibit decreased activity in enzymes sensitive to inactivation by superoxide and an accumulation of cellular succinate. **A)** Aconitase activity was measured in a spectrophotometric assay where total cellular protein was combined with NADP^+^ and isocitrate dehydrogenase and the appearance of NADPH was monitored every 30s for 5 minutes. **B)** Succinate dehydrogenase activity visualized via NBT reduction histochemistry was significantly reduced in both 293T and HEL cells without MnSOD expression. **C)** Total cellular succinate increased ~5-fold in both 293T and HEL KO cells. *p<0.01 compared to WT.

Having established and characterized suitable model systems, we next sought to determine if *SOD2*^*−/−*^ cells exhibited decreased activity in mitochondrial enzymes known to be sensitive to inactivation by O_2_^•−^. Aconitase and SDH/Complex II are key players in mitochondrial metabolism that contain Fe-S clusters susceptible to inactivation by O_2_^•−^ [20–22]. Aconitase activity was measured in a spectrophotometric assay where total cellular protein was combined with NADP^+^ and isocitrate dehydrogenase and the appearance of NADPH was measured at 340 nm. As expected, we observed a significant decrease in aconitase activity in *SOD2*^*−/−*^ HEL cells, likely due to inactivation by increased steady state levels of O_2_^•−^ (Figure 2A). In addition, SDH activity was significantly decreased in *SOD2*^*−/−*^ HEL cells (Figure 2B) as was also observed in *SOD2*^*−/−*^ HEK293 cells in a previous study [28]. Consistent with the observed decrease in SDH activity, cellular succinate levels increased ~5-fold in both 293T and HEL KO cells (Figure 2C). Taken together, these results indicate that MnSOD loss is associated with significantly decreased SDH activity, likely through O_2_^•−^-mediated disruption of Fe-S clusters in SDH subunit B, and that loss of SDH activity is co-incident with increased levels of cellular succinate, the substrate for SDH.

### Activity of TET family of demethylation enzymes was diminished in cells without MnSOD expression

**Figure 3:**
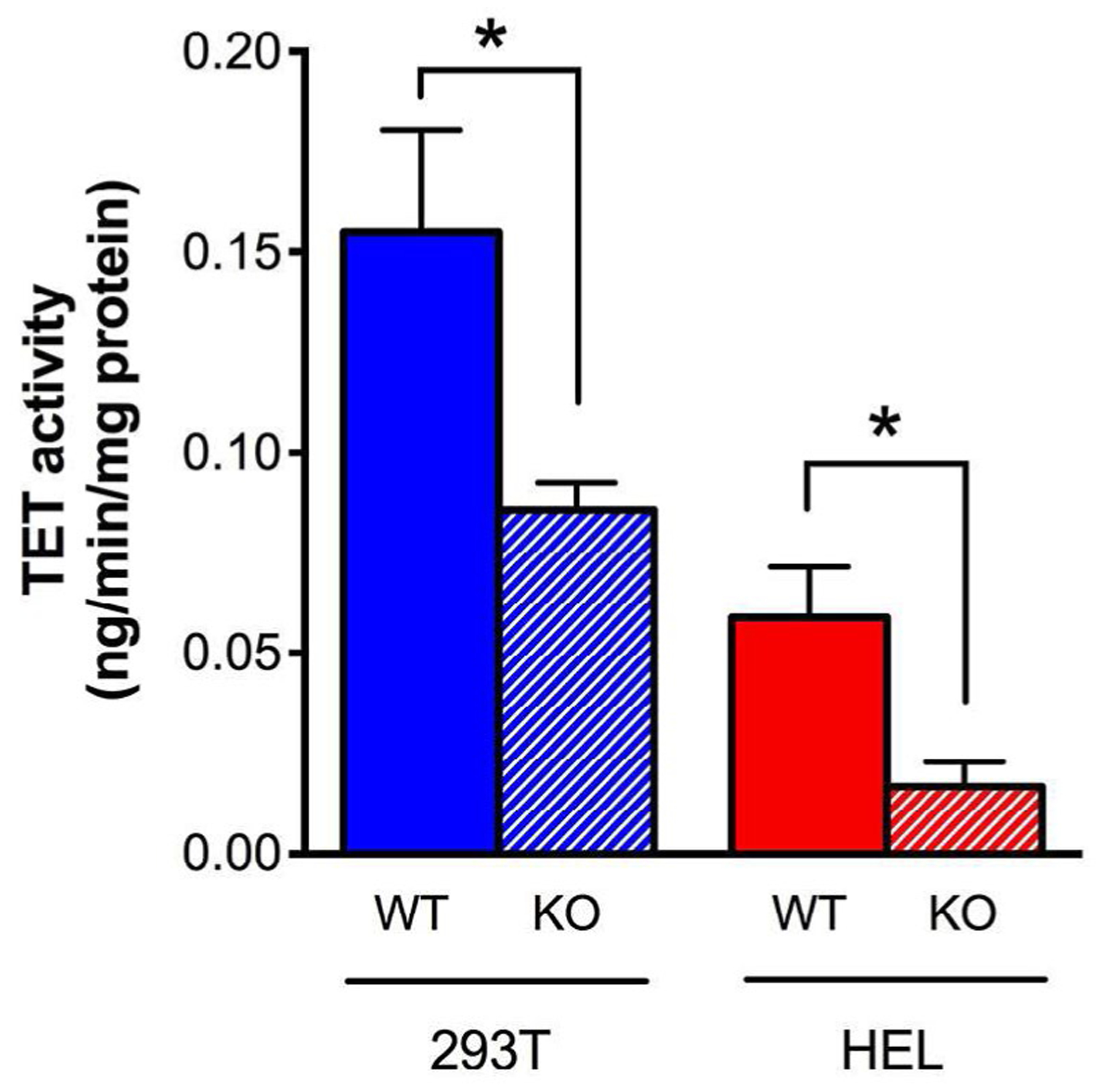
Activity of TET family of demethylation enzymes is diminished in cells lacking MnSOD. TET activity was measured in an antibody-mediated colorimetric assay using nuclear protein. A ~2-fold reduction in TET family activity was observed in MnSOD- deficient 293T cells (blue striped bar) and HEL cells (red striped bar). *p*<0.05 compared to WT.

TET DNA demethylases are nuclear enzymes that utilize α-KG and generate succinate, by which they known to be product inhibited. Therefore, we next sought to determine if the accumulation of succinate that occurs in *SOD2*^*−/−*^ cells coincided with decreased activity of TET family enzymes. To this end, TET activity was measured in an antibody-mediated colorimetric assay using nuclear protein. The results of this assay revealed a ~2-fold reduction in TET family activity in both *SOD2*^*−/−*^ 293T cells (blue striped bar) and SOD2^−/−^ HEL cells (red striped bar) (Figure 3) compared to their WT parent cell types. Overall, these results demonstrate a clear association between loss of MnSOD expression and decreased activity of TET demethylase enzymes, likely due to product-induced negative feedback inhibition resulting from elevated succinate levels.

### TET enzyme inactivation disrupted epigenetic programming and altered cell fate

**Figure 4:**
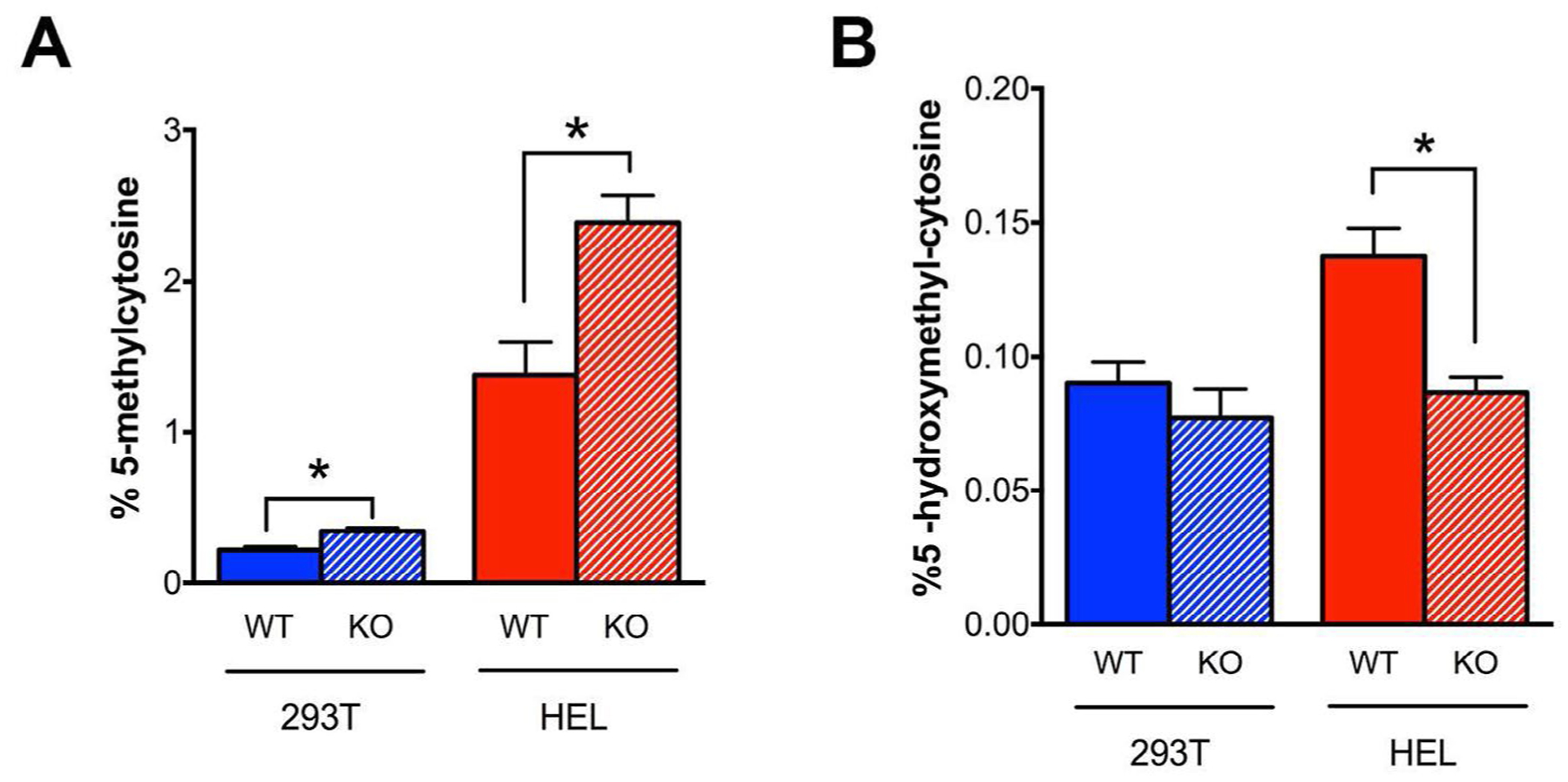
TET enzyme inactivation is associated with perturbed global epigenetic programming. **A)** An expected increase in the percentage of genomic 5-methylcytosine was observed in 293T and HEL KO cells. **B)** A corresponding decrease in the percentage of genomic 5-hydroxymethylcytosine was detected in 293T and HEL cells lacking MnSOD expression. *p<.01 compared to WT.

Based on the observation of decreased TET enzyme activity in *SOD2*^*−/−*^ cell lines, we hypothesized that this would constitute a DNA demethylation defect and that these cells would therefore display an increase in the percentage of genomic 5-mC. Indeed, we observed a statistically significant increase in percent 5-mC in both 293T and HEL *SOD2*^*−/−*^ cells compared to their respective WT parent cell lines (Figure 4A). In parallel, we observed a corresponding decrease in the percentage of the TET enzyme product 5-hmC in HEL cells lacking MnSOD expression (Figure 4B). These results agree well with the finding of decreased TET enzyme activity and demonstrate that decreased TET enzyme activity is associated with an increase in TET enzyme substrate (5-mC) and decrease in TET enzyme product (5-hmC) potentially contributing to dysregulated homeostasis of DNA methylation

The process of erythroid maturation is dependent on global DNA demethylation, which we demonstrated was disrupted in *SOD2*^*−/−*^ cells. To determine whether the DNA demethylation defect in *SOD2*^*−/−*^ cells would impair the ability of erythroid-like precursor cells to differentiate toward RBCs, we compared the ability of parent WT HEL cells to *SOD2*^*−/−*^ HEL cells to differentiate upon exposure to δ-aminolevulinic acid (δ-ALA). Traditional markers (CD235a/Glycophorin A and CD71/Transferrin receptor) were used to assess erythroid maturation of HEL cells by flow cytometry. Differentiating RBC precursors display a wave of elevated CD71 expression that diminishes upon complete maturation to RBCs, and mature human erythroid cells are CD235a-positive and CD71-negative [34,35]. At baseline, *SOD2*^*−/−*^ cells exhibited slightly higher CD71 and CD235a expression compared to WT cells.

**Figure 5:**
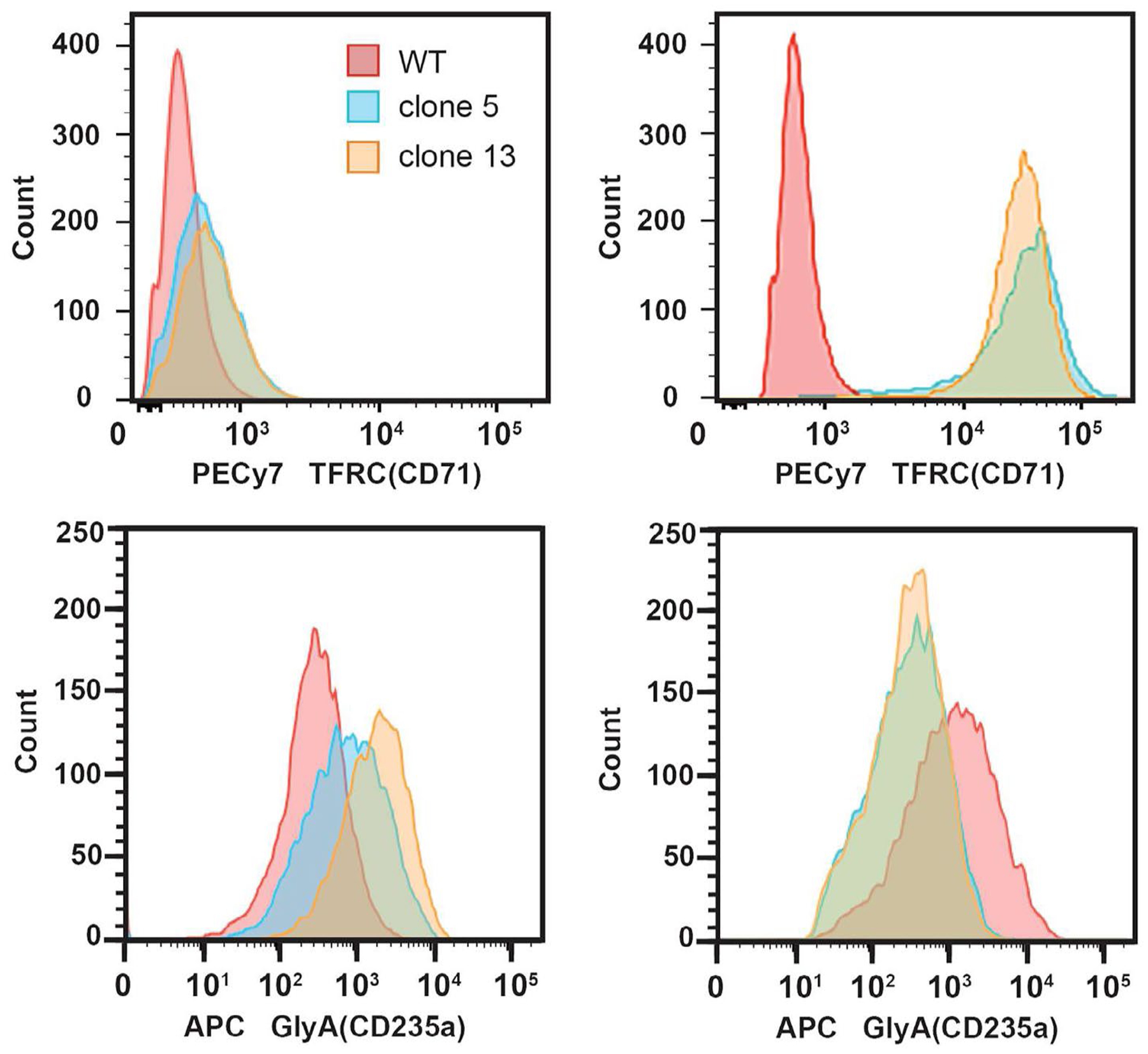
Erythroid maturation is impaired in HEL KO cells compared to WT. With the addition of δ-ALA, HEL KO cells (clone 5, blue and clone 13, orange) failed to downregulate expression of Transferrin receptor (TFRC) and decreased expression of Glycophorin A (GlyA) compared to WT cells (red) as determined by inspection of AUC.

To initiate erythroid maturation, HEL cells were grown in the presence or absence of 100 μM δ-aminolevulinic acid (δ-ALA) for 5 days. After incubation with δ-ALA for 5 days, WT HEL cells exhibited relatively low CD71 surface expression, consistent with a mature CD71-negative phenotype (Figure 5, top). By contrast, HEL *SOD2*^*−/−*^ cells (clone 5, blue and clone 13, orange) exhibited 70–80 fold increases in CD71 surface expression upon treatment with ALA demonstrating a significant defect in their ability to downregulate CD71 upon completion of the RBC maturation process (Figure 5, top). In the case of Glycophorin/CD235a, parent WT HEL cells upregulated CD235a 4-fold upon exposure to δ-ALA, consistent with normal erythroid maturation (Figure 5, bottom). In contrast, *SOD2*^*−/−*^ HEL cells exhibited a 2–4 fold decrease in CD235a (Figure 5 bottom). These results are quantified in Table 1. After 5 days in ALA, parent WT HEL cells resemble maturing erythroid cells (low TFRC, increased GlyA) whereas *SOD2*^*−/−*^ cells displayed aberrant expression of these biomarkers (high TFRC, decreased GlyA), consistent with their defect in DNA demethylation which is required for proper erythroid differentiation. Thus, *SOD2*^*−/−*^ HEL cells fail to display proper erythroid maturation. We propose that this impairment is likely due to a deficiency in TET-driven DNA demethylation.

## Discussion

**Figure 6:**
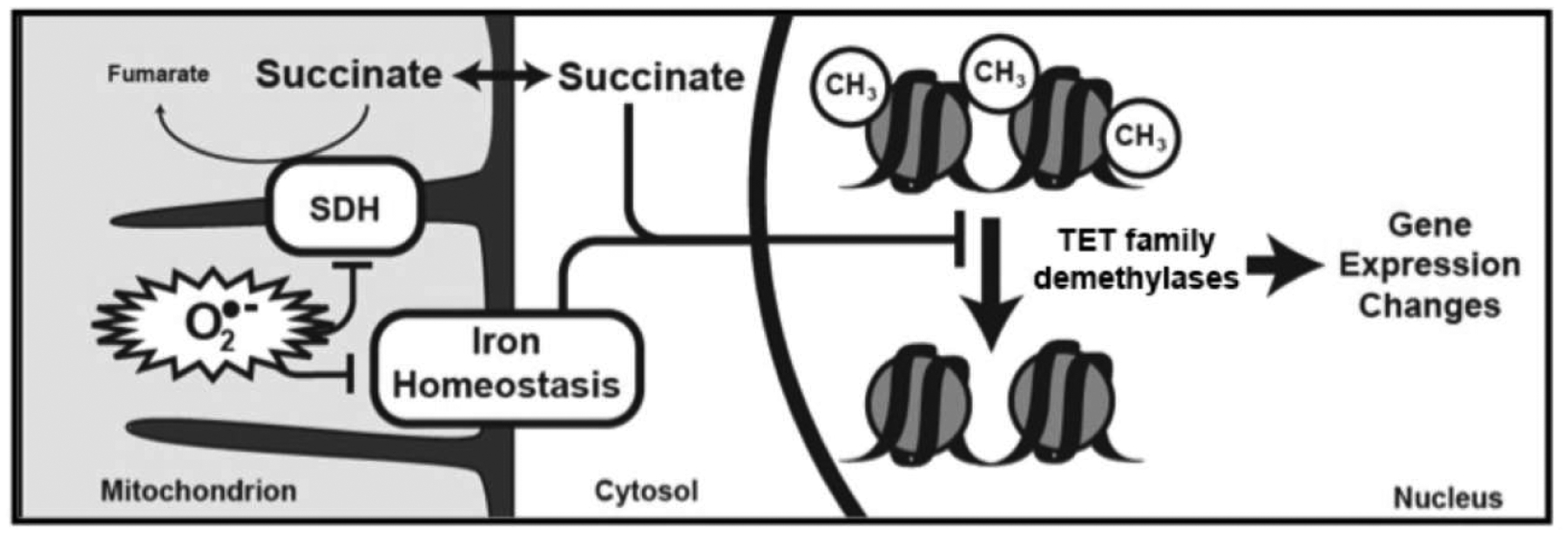
Model depicting a central role for loss of MnSOD in disrupting differentiation. Loss of MnSOD leads to higher mitochondrial superoxide which disrupts mitochondrial non-heme iron proteins including SDH resulting in elevated cellular succinate and product-inhibition of α-KG dependent TET enzymes, resulting in large scale alterations in genomic DNA methylation.

We tested the overarching hypothesis that succinate serves as a mitochondrial retrograde signal linking MnSOD activity to nuclear epigenetic reprogramming. Our results suggest a model in which increased mitochondrial O_2_^•−^ levels in cells lacking MnSOD leads to inhibition of SDH activity and accumulation of succinate. In turn, this build-up of succinate causes inactivation of the TET family of DNA demethylases. Such disruption of epigenetic homeostasis might contribute to aberrations in developmental pathways or maintenance of cell-type lineage stability. While in this study we have used erythroid differentiation in *SOD2*^*−/−*^ HEL cells as a phenotypic indicator of disrupted DNA demethylase activity, we also showed disrupted DNA demethylase activity and 5-mC levels in HEK293 cells, thus demonstrating that this effect is not specific to a single cell line or cell type. This data supports the model of an SDH/succinate/TET axis and a role for succinate as a retrograde signaling molecule of mitochondrial origin that significantly perturbs nuclear epigenetic reprogramming to affect cellular phenotype or differentiation state (Figure 6). Furthermore, our findings introduce MnSOD as a regulator of the SDH/succinate/TET axis and propose that SDH inactivation in the context of *SOD2* knockout is a result of Fe-S cluster inactivation caused by increased steady-state levels of O_2_^•−^.

Of particular interest will be the continued investigation of the MnSOD/SDH/succinate/TET axis in the context of cancer progression and dedifferentiation. The MnSOD promoter contains a CpG island that is frequently methylated in cancer cells, and MnSOD activity decreases during early carcinogenesis [36–39]. Based on this knowledge, we have proposed an “epigenetic flywheel” in which loss of SOD activity contributes to epigenetic changes, leading to further CpG island hypermethylation of the promoters of *SOD2* and tumor suppressor genes [40]. Findings in this study support the theory that decreased MnSOD activity is linked with increased DNA methylation.

Hypothetically, the epigenetic dysregulation caused by this “flywheel” effect could be initiated at any constituent of the MnSOD/SDH/TET axis. One set of initiators includes SDH mutations. Indeed, SDH mutations, are common in gastrointestinal stromal tumors and paragangliomas and are associated with decreased TET enzyme activity, DNA hypermethylation, poor differentiation, and occurrence of epithelial-to-mesenchymal transition (EMT) [17,41–45]. In addition to genetic mutations, SDH activity can be impaired by cellular iron deficiency induced by various stressors, such as lysosomal dysfunction [46,47].

Relatedly, mutations to fumarate hydratase (FH), another Fe-S cluster-containing protein, or isocitrate dehydrogenase (IDH), lead to accumulation of fumarate and D-2-hydroxyglutarate, respectively, which act in a manner similar to succinate by inhibiting TET and Jumonji histone demethylases, leading to DNA and histone hypermethylation, impairment of homologous recombination [48–50] and failure to differentiate in several contexts [5,51–53]. Interestingly, SDH and IDH mutations alone are sufficient to activate oncogenic programs in the absence of classical kinase mutations by disrupting normal chromosomal topology [54,55].

Another potential set of initiators of the proposed “flywheel” include direct mutations to TET enzymes or differential bioavailability of TET enzyme substrates and cofactors. For example, genetic loss of TET enzymes can lead to increased hematopoietic stem cell self-renewal and eventual development of a myeloproliferative phenotype [56]. Regarding TET substrates, tumor hypoxia has been shown to induce hypermethylation *via* inhibition of TET [57] and O_2_ tension determines cellular differentiation by regulating TET activity in eSCs [58]. Availability of cofactors including Fe^2+^ and ascorbate have also been shown to regulate TET enzyme activity [59–61]. Some of this knowledge is already being translated into cancer therapies. For example, ascorbate treatment has been shown to increase TET enzymatic activity and 5-hmC levels and is associated with reduced tumor progression and/orgrowth in several contexts [62–64]. It should be noted that, while we did not examine the effect of succinate accumulation of histone demethylase activity in this study, we postulate that aberrant histone methylation may also be occurring under these conditions, as KDMs are also α-KG dependent dioxygenases amenable to inhibition by succinate. Such hypotheses will be the focus to future studies.

Ultimately, our results present a link between mitochondrial oxidative metabolism and epigenetic regulation, support the role of succinate in the growing list of metabolic regulators of the epigenome, and introduce MnSOD as a modulator of succinate levels and TET enzyme activity, likely through control of SDH activity.

## Figures and Tables

**Table 1:** MnSOD deficiency inhibits erythroid differentiation in response to δ-ALA. Mean fluorescence intensities (MFI) for surface expression of markers of erythroid differentiation, transferrin receptor (CD71) and glycophorin A (CD225a). After 5 days in δ-ALA, wild type HEL cells resemble maturing erythroid cells (low TFRC, high GlyA) whereas *SOD2*^−/−^ cells displayed aberrant expression of these biomarkers, consistent with their defect in DNA demethylation which is required for erythroid differentiation. AU – arbitrary units

(MFI, AU x 10^−2^)	−δALA	+δALA	Fold Δ
	**TFRC**		
WT HEL	2.5	5.7	2.3
*SOD2*^−/−^ cl.5	4.4	31	70
*SOD2*^−/−^ cl.13	5.0	40	80
	**Glycophorin A**		
WT HEL	3.2	12	3.8
*SOD2*^−/−^ cl.5	9.0	4.5	−2.0
*SOD2*^−/−^ cl.13	20	4.5	−4.4

## References

[1] Cyr AR, Domann FE. The redox basis of epigenetic modifications: from mechanisms to functional consequences. Antioxidants & Redox signaling. 2011 Jul 15;15(2):551–89. 20919933 10.1089/ars.2010.3492 PMC3118659

[2] Janssen JJ, Grefte S, Keijer J, De Boer VC. Mito-nuclear communication by mitochondrial metabolites and its regulation by B-vitamins. Frontiers in physiology. 2019 Feb 12;10:78. 30809153 10.3389/fphys.2019.00078 PMC6379835

[3] Hitchler MJ, Domann FE. An epigenetic perspective on the free radical theory of development. Free Radical Biology and Medicine. 2007 Oct 1;43(7):1023–36. 17761298 10.1016/j.freeradbiomed.2007.06.027 PMC2981179

[4] Laukka T, Mariani CJ, Ihantola T, Cao JZ, Hokkanen J, Kaelin WG, Fumarate and succinate regulate expression of hypoxia-inducible genes via TET enzymes. Journal of Biological Chemistry. 2016 Feb 19;291(8):4256–65. 26703470 10.1074/jbc.M115.688762 PMC4759199

[5] Xiao M, Yang H, Xu W, Ma S, Lin H, Zhu H, Inhibition of α-KG-dependent histone and DNA demethylases by fumarate and succinate that are accumulated in mutations of FH and SDH tumor suppressors. Genes & Development. 2012 Jun 15;26(12):1326–38. 22677546 10.1101/gad.191056.112 PMC3387660

[6] Kaelin WG. Cancer and altered metabolism: potential importance of hypoxia-inducible factor and 2-oxoglutarate-dependent dioxygenases. InCold Spring Harbor symposia on quantitative biology 2011 Jan 1 (Vol. 76, pp. 335–345). Cold Spring Harbor Laboratory Press. 10.1101/sqb.2011.76.010975 PMC4197849 22089927

[7] Simmons JM, Müller TA, Hausinger RP. Fe II/α-ketoglutarate hydroxylases involved in nucleobase, nucleoside, nucleotide, and chromatin metabolism. Dalton Transactions. 2008(38):5132–42. 18813363 10.1039/b803512a PMC2907160

[8] Tran KA, Dillingham CM, Sridharan R. The role of α-ketoglutarate–dependent proteins in pluripotency acquisition and maintenance. Journal of Biological Chemistry. 2019 Apr 5;294(14):5408–19. 30181211 10.1074/jbc.TM118.000831 PMC6462505

[9] Lees JG, Gardner DK, Harvey AJ. Pluripotent stem cell metabolism and mitochondria: beyond ATP. Stem Cells International. 2017 Jan 1;2017. 10.1155/2017/2874283 PMC5540363 28804500

[10] Carey BW, Finley LW, Cross JR, Allis CD, Thompson CB. Intracellular α-ketoglutarate maintains the pluripotency of embryonic stem cells. Nature. 2015 Feb;518(7539):413–6. 25487152 10.1038/nature13981 PMC4336218

[11] Hwang IY, Kwak S, Lee S, Kim H, Lee SE, Kim JH, Psat1-dependent fluctuations in α-ketoglutarate affect the timing of ESC differentiation. Cell Metabolism. 2016 Sep 13;24(3):494–501. 27476977 10.1016/j.cmet.2016.06.014

[12] TeSlaa T, Chaikovsky AC, Lipchina I, Escobar SL, Hochedlinger K, Huang J, α-Ketoglutarate accelerates the initial differentiation of primed human pluripotent stem cells. Cell Metabolism. 2016 Sep 13;24(3):485–93. 27476976 10.1016/j.cmet.2016.07.002 PMC5023506

[13] Morris JP, Yashinskie JJ, Koche R, Chandwani R, Tian S, Chen CC, Baslan T, Marinkovic ZS, Sánchez-Rivera FJ, Leach SD, Carmona-Fontaine C. α-Ketoglutarate links p53 to cell fate during tumour suppression. Nature. 2019 Sep;573(7775):595–9. 31534224 10.1038/s41586-019-1577-5 PMC6830448

[14] Shearstone JR, Pop R, Bock C, Boyle P, Meissner A, Socolovsky M. Global DNA demethylation during mouse erythropoiesis in vivo. Science. 2011 Nov 11;334(6057):799–802. 22076376 10.1126/science.1207306 PMC3230325

[15] Dawlaty MM, Breiling A, Le T, Barrasa MI, Raddatz G, Gao Q, Loss of Tet enzymes compromises proper differentiation of embryonic stem cells. Developmental Cell. 2014 Apr 14;29(1):102–11. 24735881 10.1016/j.devcel.2014.03.003 PMC4035811

[16] Ko M, Bandukwala HS, An J, Lamperti ED, Thompson EC, Hastie R, Ten-Eleven-Translocation 2 (TET2) negatively regulates homeostasis and differentiation of hematopoietic stem cells in mice. Proceedings of the National Academy of Sciences. 2011 Aug 30;108(35):14566–71. 10.1073/pnas.1112317108 PMC3167529 21873190

[17] Letouzé E, Martinelli C, Loriot C, Burnichon N, Abermil N, Ottolenghi C, SDH mutations establish a hypermethylator phenotype in paraganglioma. Cancer cell. 2013 Jun 10;23(6):739–52. 23707781 10.1016/j.ccr.2013.04.018

[18] Cordes T, Wallace M, Michelucci A, Divakaruni AS, Sapcariu SC, Sousa C, Immunoresponsive gene 1 and itaconate inhibit succinate dehydrogenase to modulate intracellular succinate levels. Journal of Biological Chemistry. 2016 Jul 1;291(27):14274–84. 27189937 10.1074/jbc.M115.685792 PMC4933182

[19] Hoekstra AS, de Graaff MA, Briaire-de Bruijn IH, Ras C, Seifar RM, van Minderhout I, Inactivation of SDH and FH cause loss of 5hmC and increased H3K9me3 in paraganglioma/pheochromocytoma and smooth muscle tumors. Oncotarget. 2015 Nov 17;6(36):38777–88. 26472283 10.18632/oncotarget.6091 PMC4770736

[20] Powell CS, Jackson RM. Mitochondrial complex I, aconitase, and succinate dehydrogenase during hypoxia-reoxygenation: modulation of enzyme activities by MnSOD. American Journal of Physiology-Lung Cellular and Molecular Physiology. 2003 Jul;285(1):L189–98. 12665464 10.1152/ajplung.00253.2002

[21] Zhang Y, Marcillat O, Giulivi C, Ernster L, Davies KJ. The oxidative inactivation of mitochondrial electron transport chain components and ATPase. Journal of Biological Chemistry. 1990 Sep 25;265(27):16330–6. 2168888

[22] Gardner PR, Raineri I, Epstein LB, White CW. Superoxide radical and iron modulate aconitase activity in mammalian cells. Journal of Biological Chemistry. 1995 Jun 2;270(22):13399–405. 7768942 10.1074/jbc.270.22.13399

[23] Gardner PR. Superoxide-driven aconitase Fe–S center cycling. Bioscience Reports. 1997 Feb 1;17(1):33–42. 9171919 10.1023/a:1027383100936

[24] Case AJ, Madsen JM, Motto DG, Meyerholz DK, Domann FE. Manganese superoxide dismutase depletion in murine hematopoietic stem cells perturbs iron homeostasis, globin switching, and epigenetic control in erythrocyte precursorcells. Free Radical Biology and Medicine. 2013 Mar 1;56:17–27. 23219873 10.1016/j.freeradbiomed.2012.11.018 PMC3578015

[25] Benov L How superoxide radical damages the cell. Protoplasma. 2001 Mar 1;217(1–3):33–6. 11732335 10.1007/BF01289410

[26] Wallace MA, Liou LL, Martins J, Clement MH, Bailey S, Longo VD, Superoxide Inhibits 4Fe-4S Cluster Enzymes Involved in Amino Acid Biosynthesis Cross-compartment Protection by CuZn-superoxide dismutase. Journal of Biological Chemistry. 2004 Jul 30;279(31):32055–62. 15166213 10.1074/jbc.M403590200

[27] Li Y, Huang TT, Carlson EJ, Melov S, Ursell PC, Olson JL, Dilated cardiomyopathy and neonatal lethality in mutant mice lacking manganese superoxide dismutase. Nature Genetics. 1995 Dec;11(4):376–81. 7493016 10.1038/ng1295-376

[28] Cramer-Morales K, Heer CD, Mapuskar KA, Domann FE. SOD2 targeted gene editing by CRISPR/Cas9 yields human cells devoid of MnSOD. Free Radical Biology and Medicine. 2015 Dec 1;89:379–86. 26208779 10.1016/j.freeradbiomed.2015.07.017 PMC4890619

[29] Melov S, Coskun P, Patel M, Tuinstra R, Cottrell B, Jun AS, Mitochondrial disease in superoxide dismutase 2 mutant mice. Proceedings of the National Academy of Sciences. 1999 Feb 2;96(3):846–51. 10.1073/pnas.96.3.846 PMC15313 9927656

[30] Case AJ, McGill JL, Tygrett LT, Shirasawa T, Spitz DR, Waldschmidt TJ, Elevated mitochondrial superoxide disrupts normal T cell development, impairing adaptive immune responses to an influenza challenge. Free Radical Biology and Medicine. 2011 Feb 1;50(3):448–58. 21130157 10.1016/j.freeradbiomed.2010.11.025 PMC3026081

[31] Dorn I, Lazar-Karsten P, Boie S, Ribbat J, Hartwig D, Driller B, In vitro proliferation and differentiation of human CD34+ cells from peripheral blood into mature red blood cells with two different cell culture systems. Transfusion. 2008 Jun;48(6):1122–32. 18298595 10.1111/j.1537-2995.2008.01653.x

[32] Isla NG, Riquelme BD, Rasia RJ, Valverde JR, Stoltz JF. Quantification of glycophorin A and glycophorin B on normal human RBCs by flow cytometry. Transfusion. 2003 Aug;43(8):1145–52. 12869123 10.1046/j.1537-2995.2003.00471.x

[33] Pande A, Dorwal P, Jain D, Tyagi N, Mehra S, Sachdev R, Expression of CD71 by flow cytometry in acute leukemias: More often seen in acute myeloid leukemia. Indian Journal of Pathology and Microbiology. 2016 Jul 1;59(3):310–13. 27510666 10.4103/0377-4929.188145

[34] Marsee DK, Pinkus GS, Yu H. CD71 (transferrin receptor) an effective marker for erythroid precursors in bone marrow biopsy specimens. American Journal of Clinical Pathology. 2010 Sep 1;134(3):429–35. 20716799 10.1309/AJCPCRK3MOAOJ6AT

[35] Wangen JR, Eidenschink Brodersen L, Stolk TT, Wells DA, Loken MR. Assessment of normal erythropoiesis by flow cytometry: important considerations for specimen preparation. International Journal of Laboratory Hematology. 2014 Apr;36(2):184–96. 24118926 10.1111/ijlh.12151

[36] Huang Y, Peng J, Oberley LW, Domann FE. Transcriptional inhibition of manganese superoxide dismutase (SOD2) gene expression by DNA methylation of the 5′CpG island. Free Radical Biology and Medicine. 1997 Jan 1;23(2):314–20. 9199894 10.1016/s0891-5849(97)00095-6

[37] Huang Y, He T, Domann FE. Decreased expression of manganese superoxide dismutase in transformed cells is associated with increased cytosine methylation of the SOD2 gene. DNA and Cell Biology. 1999 Aug 1;18(8):643–52. 10463060 10.1089/104454999315051

[38] Hitchler M, Wikainapakul K, Yu L, Powers K, Attatippaholkun W, Domann F. Epigenetic regulation of manganese superoxide dismutase expression in human breast cancer cells. Epigenetics. 2006 Oct 23;1(4):163–71. 17965603 10.4161/epi.1.4.3401

[39] Dhar SK, Tangpong J, Chaiswing L, Oberley TD, Clair DK. Manganese superoxide dismutase is a p53-regulated gene that switches cancers between early and advanced stages. Cancer research. 2011 Nov 1;71(21):6684–95. 22009531 10.1158/0008-5472.CAN-11-1233 PMC3206134

[40] Cyr AR, Hitchler MJ, Domann FE. Regulation of SOD2 in cancer by histone modifications and CpG methylation: closing the loop between redox biology and epigenetics. Antioxidants & Redox Signaling. 2013 May 20;18(15):1946–55. 22946823 10.1089/ars.2012.4850 PMC3624766

[41] Killian JK, Kim SY, Miettinen M, Smith C, Merino M, Tsokos M, Succinate dehydrogenase mutation underlies global epigenomic divergence in gastrointestinal stromal tumor. Cancer Discovery. 2013 Jun 1;3(6):648–57. 23550148 10.1158/2159-8290.CD-13-0092 PMC4135374

[42] Mason EF, Hornick JL. Succinate dehydrogenase deficiency is associated with decreased 5-hydroxymethylcytosine production in gastrointestinal stromal tumors: implications for mechanisms of tumorigenesis. Modern Pathology. 2013 Nov;26(11):1492–7. 23743927 10.1038/modpathol.2013.86

[43] Malik F, Santiago T, Bahrami A, Davis E, McCarville B, Newman S, Dedifferentiation in SDH-Deficient Gastrointestinal Stromal Tumor: A Report With Histologic, Immunophenotypic, and Molecular Characterization. Pediatric and Developmental Pathology. 2019 Oct;22(5):492–8. 31072206 10.1177/1093526619846222

[44] Røsland GV, Dyrstad SE, Tusubira D, Helwa R, Tan TZ, Lotsberg ML, Epithelial to mesenchymal transition (EMT) is associated with attenuation of succinate dehydrogenase (SDH) in breast cancer through reduced expression of SDHC. Cancer & Metabolism. 2019 Dec;7(1):1–8. 31164982 10.1186/s40170-019-0197-8 PMC6544948

[45] Zhang D, Wang W, Xiang B, Li N, Huang S, Zhou W, Reduced succinate dehydrogenase B expression is associated with growth and de-differentiation of colorectal cancer cells. Tumor Biology. 2013 Aug 1;34(4):2337–47. 23645213 10.1007/s13277-013-0781-4

[46] Hughes CE, Coody TK, Jeong MY, Berg JA, Winge DR, Hughes AL. Cysteine Toxicity Drives Age-Related Mitochondrial Decline by Altering Iron Homeostasis. Cell. 2020 Jan 23;180(2):296–310. 31978346 10.1016/j.cell.2019.12.035 PMC7164368

[47] Weber RA, Yen FS, Nicholson SP, Alwaseem H, Bayraktar EC, Alam M, Maintaining iron homeostasis Is the key role of lysosomal acidity for cell proliferation. Molecular Cell. 2020 Feb 6;77(3):645–55. 31983508 10.1016/j.molcel.2020.01.003 PMC7176020

[48] Sulkowski PL, Sundaram RK, Oeck S, Corso CD, Liu Y, Noorbakhsh S, Krebs-cycle-deficient hereditary cancer syndromes are defined by defects in homologous-recombination DNA repair. Nature Genetics. 2018 Aug;50(8):1086–92. 30013182 10.1038/s41588-018-0170-4 PMC6072579

[49] Sulkowski PL, Corso CD, Robinson ND, Scanlon SE, Purshouse KR, Bai H, 2-Hydroxyglutarate produced by neomorphic IDH mutations suppresses homologous recombination and induces PARP inhibitor sensitivity. Science Translational Medicine. 2017 Feb 1;9(375). 10.1126/scitranslmed.aal2463 PMC5435119 28148839

[50] Sulkowski PL, Oeck S, Dow J, Economos NG, Mirfakhraie L, Liu Y, Oncometabolites suppress DNA repair by disrupting local chromatin signalling. Nature. 2020 Jun 3:1–6. 10.1038/s41586-020-2363-0 PMC7319896 32494005

[51] Turcan S, Rohle D, Goenka A, Walsh LA, Fang F, Yilmaz E, IDH1 mutation is sufficient to establish the glioma hypermethylator phenotype. Nature. 2012 Mar;483(7390):479–83. 22343889 10.1038/nature10866 PMC3351699

[52] Noushmehr H, Weisenberger DJ, Diefes K, Phillips HS, Pujara K, Berman BP, Identification of a CpG island methylator phenotype that defines a distinct subgroup of glioma. Cancer Cell. 2010 May 18;17(5):510–22. 20399149 10.1016/j.ccr.2010.03.017 PMC2872684

[53] Lu C, Ward PS, Kapoor GS, Rohle D, Turcan S, Abdel-Wahab O, IDH mutation impairs histone demethylation and results in a block to cell differentiation. Nature. 2012 Mar;483(7390):474–8. 22343901 10.1038/nature10860 PMC3478770

[54] Flavahan WA, Drier Y, Johnstone SE, Hemming ML, Tarjan DR, Hegazi E, Altered chromosomal topology drives oncogenic programs in SDH-deficient GISTs. Nature. 2019 Nov;575(7781):229–33. 31666694 10.1038/s41586-019-1668-3 PMC6913936

[55] Flavahan WA, Drier Y, Liau BB, Gillespie SM, Venteicher AS, Stemmer-Rachamimov AO, Insulator dysfunction and oncogene activation in IDH mutant gliomas. Nature. 2016 Jan;529(7584):110–4. 26700815 10.1038/nature16490 PMC4831574

[56] Moran-Crusio K, Reavie L, Shih A, Abdel-Wahab O, Ndiaye-Lobry D, Lobry C, Tet2 loss leads to increased hematopoietic stem cell self-renewal and myeloid transformation. Cancer Cell. 2011 Jul 12;20(1):11–24. 21723200 10.1016/j.ccr.2011.06.001 PMC3194039

[57] Thienpont B, Steinbacher J, Zhao H, D’Anna F, Kuchnio A, Ploumakis A, Tumour hypoxia causes DNA hypermethylation by reducing TET activity. Nature. 2016 Sep;537(7618):63–8. 27533040 10.1038/nature19081 PMC5133388

[58] Burr S, Caldwell A, Chong M, Beretta M, Metcalf S, Hancock M, Oxygen gradients can determine epigenetic asymmetry and cellular differentiation via differential regulation of Tet activity in embryonic stem cells. Nucleic Acids Research. 2018 Feb 16;46(3):1210–26. 29186571 10.1093/nar/gkx1197 PMC5814828

[59] Zhao B, Yang Y, Wang X, Chong Z, Yin R, Song SH, Redox-active quinones induces genome-wide DNA methylation changes by an iron-mediated and Tet-dependent mechanism. Nucleic Acids Research. 2014 Feb 1;42(3):1593–605. 24214992 10.1093/nar/gkt1090 PMC3919571

[60] Shenoy N, Bhagat T, Nieves E, Stenson M, Lawson J, Choudhary GS, Upregulation of TET activity with ascorbic acid induces epigenetic modulation of lymphoma cells. Blood Cancer Journal. 2017 Jul;7(7):e587-. 28731456 10.1038/bcj.2017.65 PMC5549257

[61] Minor EA, Young JI, Wang G. Ascorbate induces ten-eleven translocation (Tet) methylcytosine dioxygenase-mediated generation of 5-hydroxymethylcytosine. Journal of Biological Chemistry. 2013 May 10;288(19):13669–74. 23548903 10.1074/jbc.C113.464800 PMC3650403

[62] Shenoy N, Bhagat TD, Cheville J, Lohse C, Bhattacharyya S, Tischer A, Ascorbic acid–induced TET activation mitigates adverse hydroxymethylcytosine loss in renal cell carcinoma. The Journal of Clinical Investigation. 2019 Apr 1;129(4):1612–25. 30702441 10.1172/JCI98747 PMC6436862

[63] Cimmino L, Dolgalev I, Wang Y, Yoshimi A, Martin GH, Wang J, Restoration of TET2 function blocks aberrant self-renewal and leukemia progression. Cell. 2017 Sep 7;170(6):1079–95. 28823558 10.1016/j.cell.2017.07.032 PMC5755977

[64] Ge G, Peng D, Xu Z, Guan B, Xin Z, He Q, Restoration of 5-hydroxymethylcytosine by ascorbate blocks kidney tumour growth. EMBO Reports. 2018 Aug;19(8):e45401. 29959161 10.15252/embr.201745401 PMC6073201

